# Potential common molecular mechanisms between Sjögren syndrome and inclusion body myositis: a bioinformatic analysis and *in vivo* validation

**DOI:** 10.3389/fimmu.2023.1161476

**Published:** 2023-04-21

**Authors:** Li Zeng, Kai Chen, Feng Xiao, Chun-yan Zhu, Jia-ying Bai, Song Tan, Li Long, Yi Wang, Qiao Zhou

**Affiliations:** ^1^ Department of Neurology, Sichuan Academy of Medical Science and Sichuan Provincial People’s Hospital, University of Electronic Science and Technology of China, Chengdu, China; ^2^ Sichuan Provincial Key Laboratory for Human Disease Gene Study, Sichuan Provincial People's Hospital, Chengdu, China; ^3^ Department of Rheumatology and Immunology, Sichuan Academy of Medical Science and Sichuan Provincial People’s Hospital, University of Electronic Science and Technology of China, Chengdu, China; ^4^ Department of Critical Care Medicine, Sichuan Academy of Medical Science and Sichuan Provincial People’s Hospital, University of Electronic Science and Technology of China, Chengdu, China; ^5^ Clinical Immunology Translational Medicine Key Laboratory of Sichuan Province, Sichuan Provincial People’s Hospital, University of Electronic Science and Technology of China, Chengdu, China

**Keywords:** Sjögren syndrome, inclusion body myositis, bioinformatic analysis, antigen processing and presentation, viral infection, immune infiltration landscape

## Abstract

**Background:**

Inclusion body myositis (IBM) is a slowly progressive inflammatory myopathy that typically affects the quadriceps and finger flexors. Sjögren’s syndrome (SS), an autoimmune disorder characterized by lymphocytic infiltration of exocrine glands has been reported to share common genetic and autoimmune pathways with IBM. However, the exact mechanism underlying their commonality remains unclear. In this study, we investigated the common pathological mechanisms involved in both SS and IBM using a bioinformatic approach.

**Methods:**

IBM and SS gene expression profiles were obtained from the Gene Expression Omnibus (GEO). SS and IBM coexpression modules were identified using weighted gene coexpression network analysis (WGCNA), and differentially expressed gene (DEG) analysis was applied to identify their shared DEGs. The hidden biological pathways were revealed using Gene Ontology (GO) and Kyoto Encyclopedia of Genes and Genomes (KEGG) analysis. Furthermore, protein−protein interaction (PPI) networks, cluster analyses, and hub shared gene identification were conducted. The expression of hub genes was validated by reverse transcription quantitative polymerase chain reaction (RT−qPCR). We then analyzed immune cell abundance patterns in SS and IBM using single-sample gene set enrichment analysis (ssGSEA) and investigated their association with hub genes. Finally, NetworkAnalyst was used to construct a common transcription factor (TF)-gene network.

**Results:**

Using WGCNA, we found that 172 intersecting genes were closely related to viral infection and antigen processing/presentation. Based on DEG analysis, 29 shared genes were found to be upregulated and enriched in similar biological pathways. By intersecting the top 20 potential hub genes from the WGCNA and DEG sets, three shared hub genes (*PSMB9*, *CD74*, and *HLA-F*) were derived and validated to be active transcripts, which all exhibited diagnostic values for SS and IBM. Furthermore, ssGSEA showed similar infiltration profiles in IBM and SS, and the hub genes were positively correlated with the abundance of immune cells. Ultimately, two TFs (HDGF and WRNIP1) were identified as possible key TFs.

**Conclusion:**

Our study identified that IBM shares common immunologic and transcriptional pathways with SS, such as viral infection and antigen processing/presentation. Furthermore, both IBM and SS have almost identical immune infiltration microenvironments, indicating similar immune responses may contribute to their association.

## 1 Introduction

Inclusion body myositis affects mostly people over 45 years of age and has distinctive clinical and pathologic characteristics. Muscle weakness is slow-progressing, frequently asymmetrical and predominantly affects the quadriceps and finger flexors. The histopathological features of IBM are complex and consist of not only lymphocytic infiltration (primarily CD8 T cells) but also congophilic inclusions, rimmed vacuoles and mitochondrial changes (1). Several mechanisms have been proposed in the pathogenesis of IBM, including autoimmunity and muscular degeneration (2–4). However, its exact pathogenic mechanism is unclear at present, and effective treatments are still lacking (3).

Sjögren’s syndrome (SS) is an autoimmune disorder characterized by lymphocytic infiltration of exocrine glands, primarily salivary glands and lacrimal glands (5). Except for exocrine gland manifestations, patients may also develop extraglandular features, resulting in the involvement of most organ systems, including muscles (5). Since 1982, a few case reports have described the coexistence of IBM in patients with SS (6–11). A study from Greece found that three out of 518 SS patients (0.6%) suffered from IBM, which is much higher than its prevalence in the general population (24.8-45.6 individuals per million) (12, 13). Similarly, a prospective cohort study carried out in France followed 395 patients with SS, finding four diagnosed with myositis, of whom two developed IBM (0.5%) (14). Interestingly, even though there were no clinical IBM findings, 8/36 SS patients showed muscular histopathological features that resembled those of IBM, suggesting a specific predisposition to IBM among SS patients (15). On the other hand, the prevalence of concurrence SS in IBM patients was reported to be 6% in United States, whereas it was just 0.1-0.6% in the general population

(16, 17). All these findings suggest that SS and IBM may share common pathogenic pathways, and currently, three possible mechanisms have been reported. The first is shared autoantibodies. Anti-SSA antibodies, which are the primary serological marker of SS, were detected in 20% of IBM patients, while anti-cN1A antibodies, a valuable biomarker for IBM, were also found in 12% of SS patients (2, 18, 19). The second is related to genetic susceptibility. The HLA-B8, DRB1*0301 (DR3) haplotype has consistently been reported to be associated with IBM in 60-75% of cases (20). Additionally, a study of 57 Australian cases found that the HLA-DRB1*0301 (DR3) allele was linked to decreased quadriceps muscle strength and faster decline in strength (21). A similar association has also been found between SS and HLA-A1, B8 and DR3 (22). Notably, a clinical study reported that six patients with both IBM and SS had the same HLA-DR3 and major histocompatibility complex (MHC) 8.1 ancestral haplotype, suggesting a common genetic predisposition associated with MHC (23). The third one is the expansion of clonal T cells has been demonstrated in both muscle biopsy samples from IBM patients (15/15) (24) and exocrine gland samples from SS patients (25). Nevertheless, these studies mainly focused on clinical or basic experiments, and few explored the whole genetic background for SS or IBM. An analysis of the transcriptional profile shared by SS and IBM may reveal their common pathogenesis.

**Figure 1:**
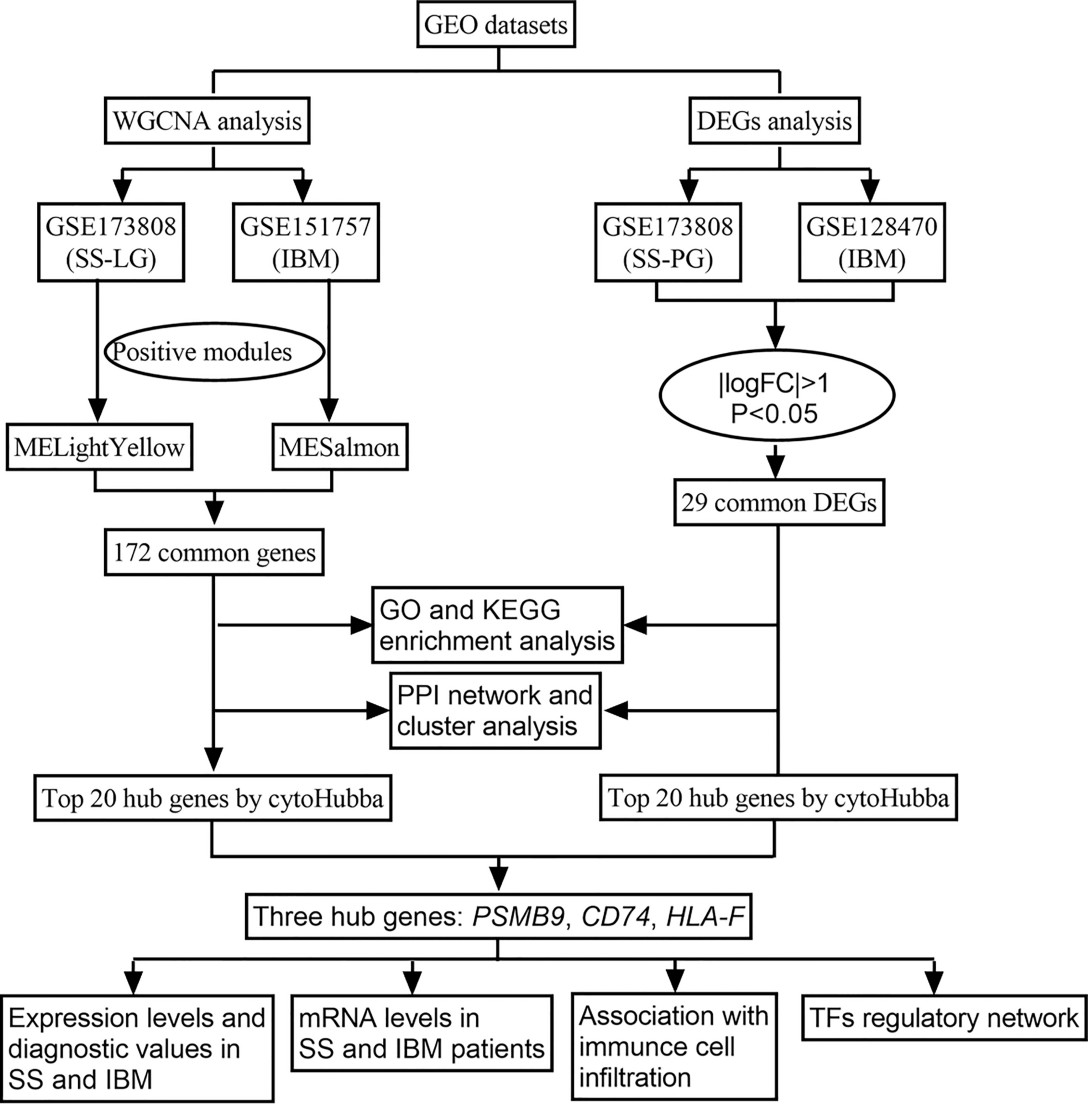
Flow chart of the research.

With the advent of bioinformatics and high-throughput sequencing, researchers have been able to analyze data on thousands of genes quickly and gain a better understanding of disease pathogenesis from a transcriptional perspective. In this study, we used weighted gene coexpression network analysis (WGCNA) to identify coexpression modules between SS and IBM and analyze differentially expressed genes (DEGs) to identify shared DEGs in a second cohort. Then, we performed functional enrichment analysis to explore common pathways. Furthermore, we performed protein−protein interaction (PPI) networks and cluster analyses to identify gene modules and shared hub genes. Reverse transcription quantitative polymerase chain reaction (RT−qPCR) was applied to validate the expression of shared hub genes. **Figure 1** shows the flowchart for the research. Through an integrated bioinformatic approach and *in vivo* validation, this study is the first to investigate the common pathological mechanisms between SS and IBM.

## 2 Materials and methods

### 2.1 GEO dataset collection

Using the keywords “Sjögren syndrome” and “inclusion body myositis”, we searched the GEO databank for SS and IBM high-throughput sequencing and expression microarray data (Gene Expression Omnibus, https://www.ncbi.nlm.nih.gov/geo/). Three datasets (GSE173808, GSE151757 and GSE128470) were downloaded from the GEO database. GSE173808 is an SS dataset, while GSE151757 and GSE128470 are IBM datasets. Since GSE173808 contains high-throughput sequencing data from both labial glands (LG) and parotid glands (PG), we divided it into two parts, GSE173808-LG and GSE173808-PG. **Table 1** summarizes the detailed information of the four datasets. GSE173808-LG and GSE151757 were paired for WGCNA, while GSE173808-PG and GSE128470 were paired for DEG analysis.

**Table 1:** Detailed information of GEO datasets containing the SS and IBM patients.

	GSE Number	Platform	Samples	Organism	Source types	Group
1	GSE173808-LG	GPL16791	38 SS and 19 controls	Homo sapiens	Labial gland	WGCNA analysis
2	GSE151757	GPL16791	24 IBM and 9 controls	Homo sapiens	Muscle	WGCNA analysis
3	GSE173808-PG	GPL16791	37 SS and 20 controls	Homo sapiens	Parotid gland	DEG analysis
4	GSE128470	GPL96	26 IBM and 12 controls	Homo sapiens	Muscle	DEG analysis
5	Local cohort	–	16 SS and 10 controls	Homo sapiens	Peripheral blood	Validation
6	Local cohort	–	13 IBM and 10 controls	Homo sapiens	Muscle	Validation

SS, Sjögren syndrome; IBM, inclusion body myositis; GEO, Gene Expression Omnibus.

### 2.2 WGCNA

To obtain SS- and IBM-related modules, the WGCNA package in R (26) was applied to the GSE173808-LG and GSE151757 datasets. First, we calculated the variance for each gene expression value and filtered out genes with absolute deviations greater than 25% from the median. Then, we used the goodSampleGenes function to eliminate samples with outlier characteristics (**Supplementary Figures 1A, B** **)**. Subsequently, the “pickSoftThreshold” function was used to determine an appropriate soft threshold to build a scale-free network, and the soft threshold β was 14 in SS and 12 in IBM (**Supplementary Figures 2A, B** **)**. Afterward, a hierarchical clustering dendrogram was further constructed, and similar genes were separated into different modules with at least 30 genes. Analogous modules were consolidated again according to MEDissThres (module eigengenes dissimilarity threshold) = 0.2. Finally, we used Pearson correlation analysis to determine the correlation between modules and disease phenotypes of interest. Our analysis focused on modules that had high correlations with the phenotypic interests, and we selected genes from disease-related modules for further analysis.

### 2.3 Identification of intersecting genes in SS and IBM *via* WGCNA

Based on the module trait correlation and the *P* value of eigengenes and phenotypic traits of each module, we selected the modules that were highly associated with SS and IBM. Then, we identified 172 intersecting genes in modules positively related to SS and IBM using the Jvenn online tool in http://jvenn.toulouse.inra.fr/app/example.html (27).

### 2.4 Detection of shared DEGs in SS and IBM

Other SS and IBM datasets (GSE173808-PG and GSE128470) were analyzed for gene expression differentiation. The “DESeq2” package in R software was used to identify DEGs in the GSE173808-PG dataset, while the “limma” package was used to identify DEGs in the GSE128470 dataset. Log_2_|fold change (FC)|>1 and *P* value<0.05 were the screening criteria. Shared DEGs between the SS and IBM databases were revealed using Jvenn (27).

### 2.5 GO and KEGG enrichment analysis

Gene Ontology (GO) and Kyoto Encyclopedia of Genes and Genomes (KEGG) analyses of common genes were completed in R using the “enrichplot” and “clusterProfiler” packages to identify biological functions and pathways. P < 0.05 was recognized as a significant term/pathway.

### 2.6 PPI network construction and cluster analysis

We constructed the PPI network utilizing the Search Tool for Retrieval of Interacting Genes (STRING; http://string-db.org). PPI networks were visualized with Cytoscape (version 3.7.2) with a minimum interaction score > 0.4. Subsequently, we performed cluster analysis using the MCODE algorithm from the Cytoscape plug-in with default parameters.

### 2.7 Screening and validation of shared hub genes

We used the maximal clique centrality (MCC) algorithm from the cytoHubba plug-in to identify hub genes in PPI networks with high connectivity. We identified three shared hub genes using the Jvenn online tool (27) by intersecting the top 20 hub genes from WGCNA with the top 20 hub genes from the DEG analysis. Then, hub shared gene expression was verified in the four datasets with the help of GraphPad Prism software (version 9.3). Furthermore, with the R package “pROC”, we created receiver operating characteristic (ROC) curves to assess the diagnostic power of the shared hub genes.

### 2.8 Clinical samples

Between 2019 and 2022, sixteen patients with SS diagnosed according to the American College of Rheumatology/European League Against Rheumatism 2016 criteria and ten healthy donors were included (28). Patients with comorbidities such as hypertension, diabetes, hyperlipidemia, chronic inflammatory disease, other autoimmune diseases, or cancer were excluded. For each participant, 1 ml peripheral fasting blood was collected into a 5 ml ethylene diamine tetra-acetic acid (EDTA) tube. At the same time, thirteen patients with IBM were diagnosed according to the inclusion body myositis workshop of the European Neuromuscular Centre (ENMC) 2011 criteria (4). Ten muscle tissues with no histopathological changes were used as controls. All muscle samples were cryopreserved at -80°C for subsequent testing. The study was conducted in accordance with the Declaration of Helsinki and was approved by the Ethical Committee of Sichuan Provincial People’s Hospital. All participants signed informed consent forms.

### 2.9 Reverse transcription−quantitative polymerase chain reaction analysis

Total RNA from the skeletal muscle tissue and whole blood was isolated using TRIzol reagent (Invitrogen; Thermo Fisher Scientific, Inc.) and reverse transcribed (High-Capacity RNA-to-cDNA kit, Applied Biosystems, Foster City, CA). The primer sequences were PSMB9 forward, 5’−CGCTTCACCACAGACGCTAT-3’ and reverse 5’- TGCCCAAGATGACTCGATGG-3’; CD74 forward, 5’- AGAACCTGCAGCTGGAGAAC-3’ and reverse 5’-GGGTCAGCATTCCCCTGG-3’; HLA-F forward 5’-TGGCCTTGTTGTCCTTGGAG-3’ and reverse 5’- AGAAGACAGTCCTCCCTGAGA-3’; and GAPDH forward 5’- CACTAGGCGCTCACTGTTCT-3’ and reverse 5’- GCCCAATACGACCAAATCCGT-3’. The RT−qPCR analysis was performed on an ABI 7900 system using Applied Biosystems Power SYBR Green PCR Master mix (Thermo Fisher Scientific, Inc.) in triplicate. In brief, 0.5 μM forward and reverse primers, 2 μl of buffer and 100 ng of cDNA templates were added to each tube, and the total volume was adjusted to 20 μl with RNase-free water (Thermo Fisher Scientific Inc.). PCR activation was at 95°C for 20 s, followed by 40 cycles of 1 s at 95°C to denature and 20 s at 60°C to extend. Reactions were performed in triplicate, and relative changes in target gene expression were normalized to the expression levels of GAPDH and calculated using the 2^–△△Ct^ method.

### 2.10 Assessment of immune cell abundance in SS and IBM

We assessed the levels of 28 immune cells in SS and IBM patients using a single-sample gene set enrichment analysis (ssGSEA) algorithm in the R package (29) in GSE173808-LG and GSE151757 datasets. The expression levels of different immune infiltrating cells between disease group and control were compared using the Wilcoxon rank-sum test (*P*<0.05) and displayed in boxplots. A Spearman correlation analysis was conducted between the 28 immune cells and the hub shared genes using the “ggplot2” package.

### 2.11 Prediction of transcription factors associated with shared hub genes

We constructed the TF-gene interactions of the shared hub genes with the NetworkAnalyst tool (v2019; https://www.networkanalyst.ca/). The shared hub genes and TFs were plotted using Cytoscape.

### 2.12 Statistical analysis

All data are presented as the mean and standard deviation. The gene expression levels were calculated using Student’s t−test by GraphPad Prism™ software (version 9.0; GraphPad Software Inc., La Jolla, CA, USA). *P*<0.05 was considered to indicate a statistically significant difference.

## 3 Results

### 3.1 Identification of shared gene signatures in SS and IBM *via* WGCNA

**Figure 2:**
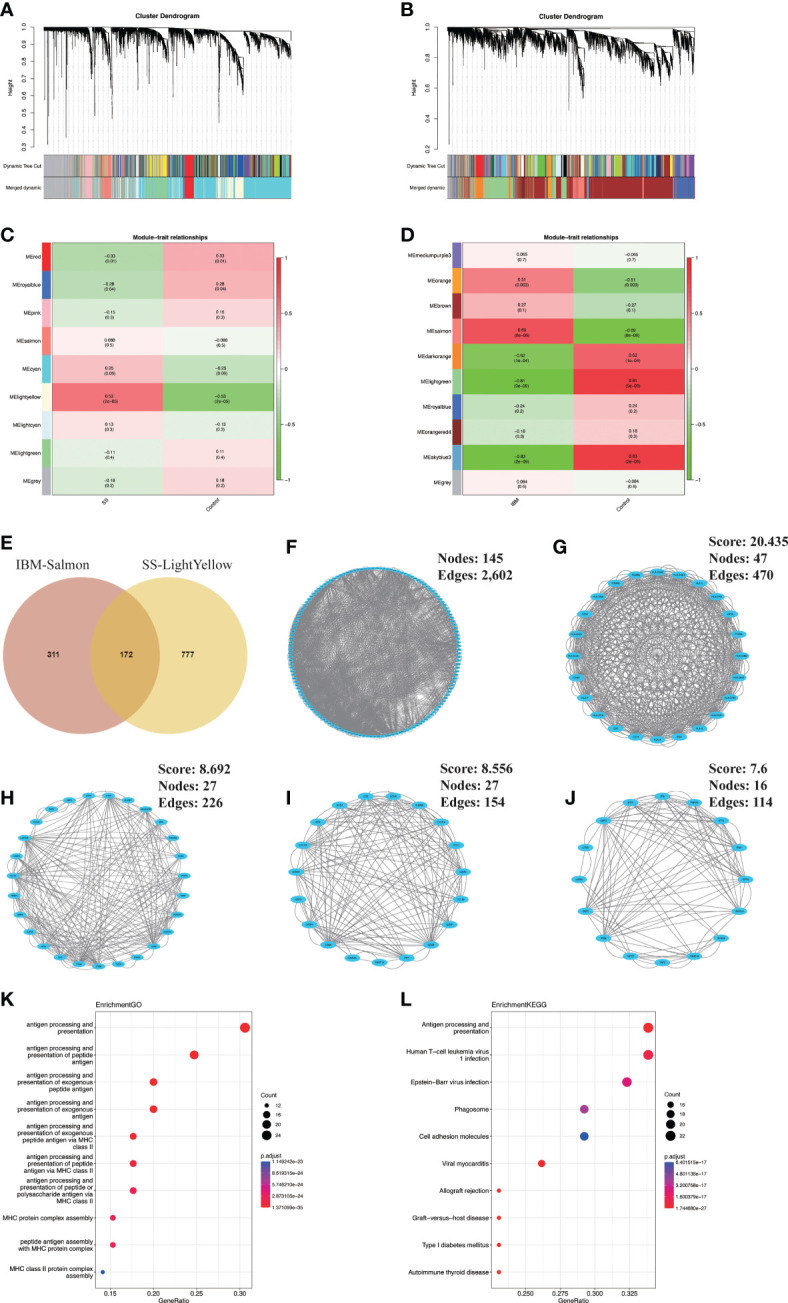
Identification and analysis of shared gene signatures in SS and IBM *via* WGCNA. **(A–D)** WGCNA of the GSE173808 and GSE151757 datasets. **(A)** The coexpression gene cluster dendrogram in SS. **(B)** The coexpression gene cluster dendrogram in IBM. **(C)** Correlation analysis between module genes and clinical phenotypes in SS. **(D)** Correlation analysis between module genes and clinical phenotypes in IBM. **(E)** Venn diagram for intersecting genes between the light-yellow module of SS and the salmon module of IBM. **(F)** The PPI network of the intersecting genes. **(G–J)** Four clusters extracted by MCODE. **(K)** The top 10 GO terms of four gene clusters. **(L)** The top 10 KEGG pathways of four gene clusters. WGCNA, weighted gene coexpression network analysis; SS, Sjögren syndrome; IBM, inclusion body myositis. PPI, protein−protein interaction; MCODE, Minimal Common Oncology Data Elements; GO, Gene Ontology; KEGG, Kyoto Encyclopedia of Genes and Genomes.

In GSE173808-LG, nine modules were identified *via* WGCNA, each represented by a different color. Then, we evaluated the disease-module association by creating a heatmap based on the Spearman correlation coefficient (**Figures 2A–C**). Module “MElightyellow” exhibited a high correlation with SS and was categorized as an SS-related module (r = 0.53, *p* = 2e-05). A total of 949 genes from the MElightyellow module were further used for analysis. Similarly, we obtained ten modules from the GSE151757 dataset using WGCNA, and four modules, “MEsalmon”, “MEdarkorange”, “MElightgreen” and “MEskyblue3”, had high correlations with IBM (MEsalmon: r = 0.69, *p* = 8e-06; MEdarkorange: r = -0.62, *p* = 1e-04; MElightgreen: r = -0.81, *p* = 9e-09; MEskyblue3: r = -0.83, *p* = 2e-09) (**Figures 2B–D**). MEsalmon was positively correlated with IBM, while MEdarkorange, MElightgreen and MEskyblue3 were negatively correlated. The positively correlated module MEsalmon, containing 483 genes, was categorized as an IBM-related module (**Figure 2D**). A total of 172 common genes were screened from the intersection of SS positively related gene modules (MElightyellow module) and IBM positively related gene modules (MEsalmon module) (**Figure 2E**, **Supplementary Table 1**). Furthermore, a PPI network of intersecting genes was constructed using the STRING database. By removing discrete proteins, we obtained a network of 145 nodes and 2,602 edges (**Figure 2F**, **Supplementary Table 2**). We then applied the MCODE plug-in to extract four closely connected gene cluster modules (**Figures 2G–J**). To investigate the potential biological functions of these genes, we performed functional enrichment analysis on these four clusters. According to GO analysis results, these genes were mainly associated with antigen processing and presentation and MHC protein complex assembly (**Figure 2K**). Based on KEGG analysis results, the gene set had significant enrichment in antigen processing and presentation as well as microbial infections (e.g., human T-cell leukemia virus 1 infection, Epstein−Barr virus infection and viral myocarditis) (**Figure 2L**).

### 3.2 Validation of shared gene signatures in SS and IBM *via* DEG analysis

**Figure 3:**
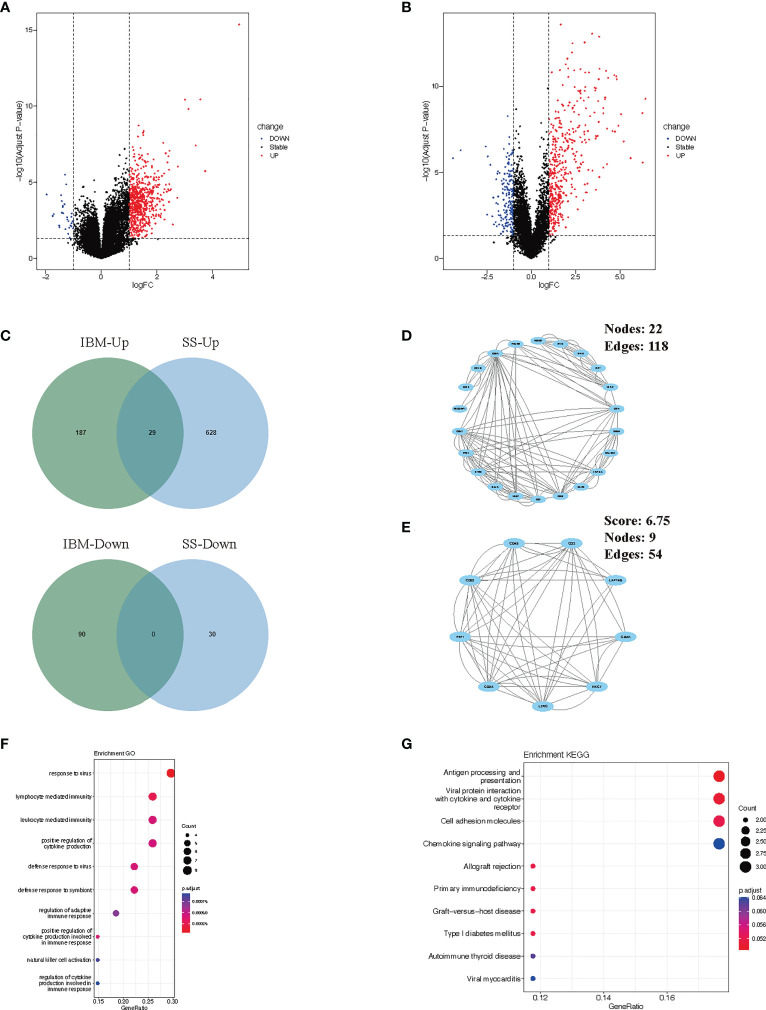
Verification and analysis of shared DEGs in SS and IBM. **(A)** Volcano plots of the DEGs in SS. **(B)** Volcano plots of the DEGs in IBM. **(C)** Venn diagram for shared DEGs in the GSE173808 and GSE128470 datasets. **(D)** The PPI network of the shared DEGs. **(E)** One cluster extracted by the MCODE plug-in. **(F)** GO enrichment results of shared DEGs. **(G)** KEGG enrichment pathways of shared genes. DEGs, differentially expressed genes; SS, Sjögren syndrome; IBM, inclusion body myositis. PPI, protein−protein interaction; MCODE, Minimal Common Oncology Data Elements; GO, Gene Ontology; KEGG, Kyoto Encyclopedia of Genes and Genomes.

To verify our findings, we analyzed the differential gene expression of the GSE173808-PG and GSE128470 datasets. We identified 687 DEGs (657 upregulated and 30 downregulated genes) in the GSE173808-PG dataset, while 306 DEGs (216 upregulated and 90 downregulated genes) were screened out in the GSE173808-PG dataset. Volcano plots were used to visualize the DEGs in both datasets (**Figures 3A, B** **)**. Using a Venn diagram, we identified 29 upregulated genes that were shared between GSE173808-PG and GSE128470 (**Figure 3C**). Furthermore, a PPI network was constructed, which had 22 nodes and 118 edges after discrete proteins were removed (**Figure 3D**). A cluster with 9 nodes and 54 edges was then extracted using algorithms from the MCODE plug-in (**Figure 3E**). The same GO and KEGG enrichment analyses that were conducted in the WGCNA was also performed on the DEGs in IBM and SS. According to GO analysis, intersecting genes were primarily associated with response to virus, lymphocyte and leukocyte mediated immunity, and regulation of immunity (**Figure 3F**). Based on KEGG analysis, the gene set had significant enrichment in antigen processing and presentation as well as microbial infections (e.g., viral protein interaction with cytokine and cytokine receptor, viral myocarditis) (**Figure 3G**). In accordance with the WGCNA results, “antigen processing and presentation” and “viral infection” were enriched again.

### 3.3 Shared hub gene screening and *in vivo* validation in SS and IBM patients

**Figure 4:**
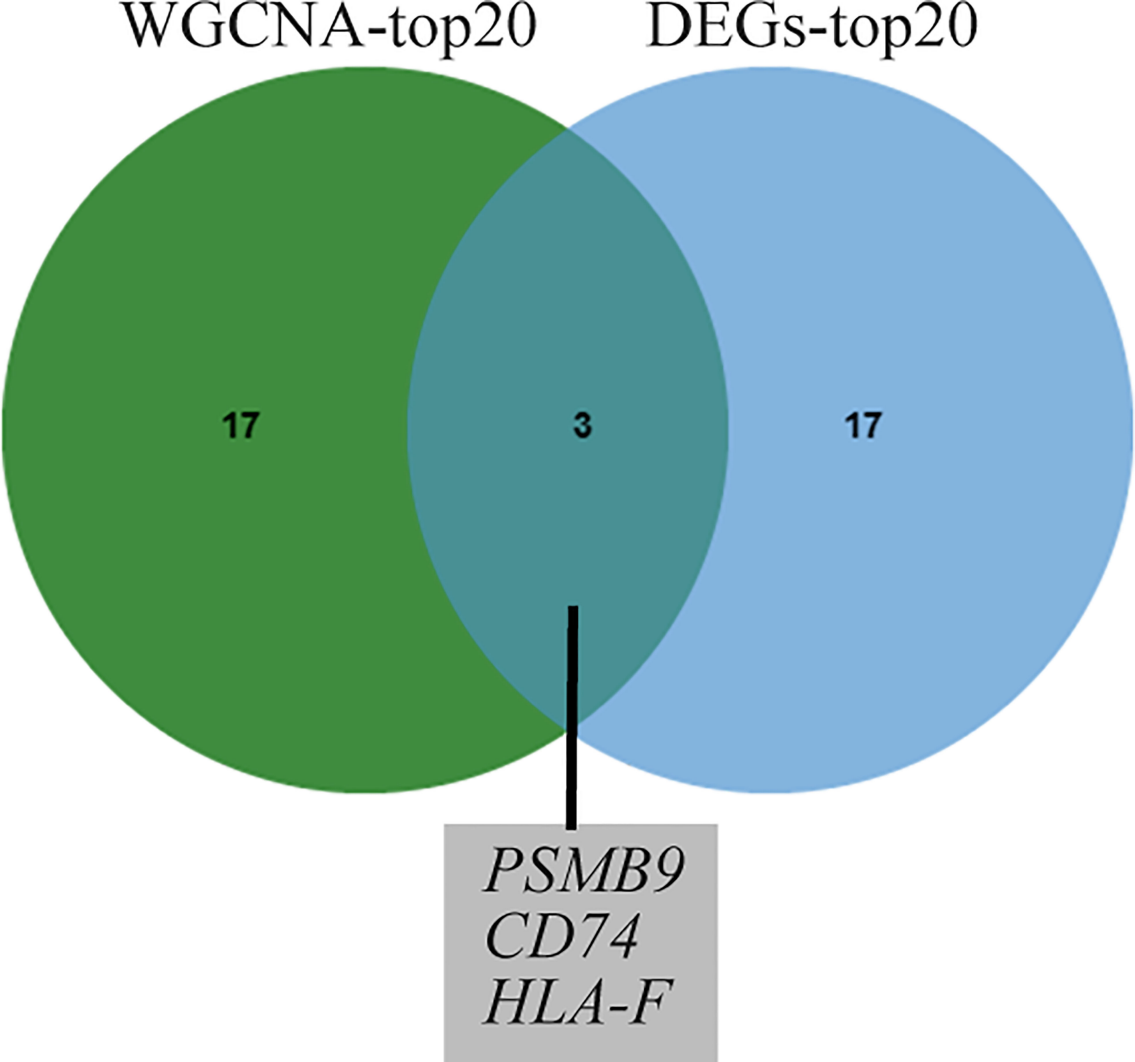
Venn diagram of common genes from the top 20 hub genes of WGCNA and DEG analysis. WGCNA, weighted gene coexpression network analysis; DEGs, differentially expressed genes.

**Figure 5:**
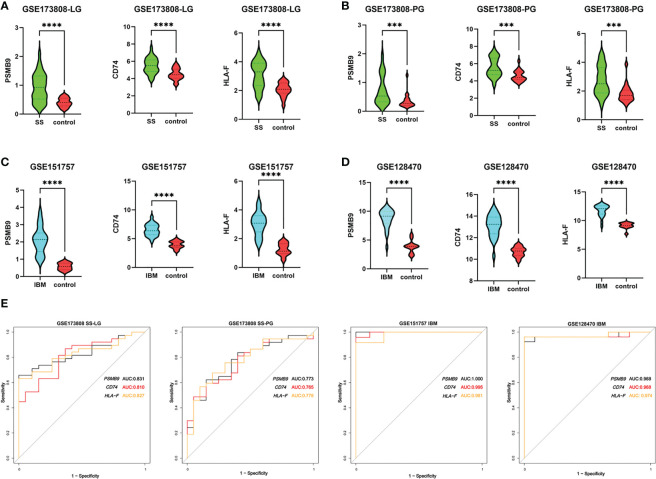
Expression levels and diagnostic values of hub genes. **(A)** The expression of *PSMB9*, *CD74* and *HLA-F* in labial gland tissues of SS (n=38) compared with controls (n=19) in the GSE173808 dataset. **(B)** The expression of *PSMB9*, *CD74* and *HLA-F* in parotid gland tissue samples of SS patients (n=37) compared with controls (n=20) in the GSE173808 dataset. **(C)** The expression of *PSMB9*, *CD74* and *HLA-F* in muscle samples of IBM patients (n=24) compared with controls (n=9) in the GSE151757 dataset. **(D)** The expression of *PSMB9*, *CD74* and *HLA-F* in muscle samples of IBM patients (n=26) compared with controls (n=12) in the GSE128470 dataset. The two groups were compared using a nonparametric Student’s t test with a *P* value of 0.05. ***p<0.001; ****p<0.0001; **(E)** The ROC curves of *PSMB9*, *CD74* and *HLA-F* in the four datasets. SS, Sjögren syndrome; IBM, inclusion body myositis. LG, labial gland; PG, parotid gland.

**Figure 6:**
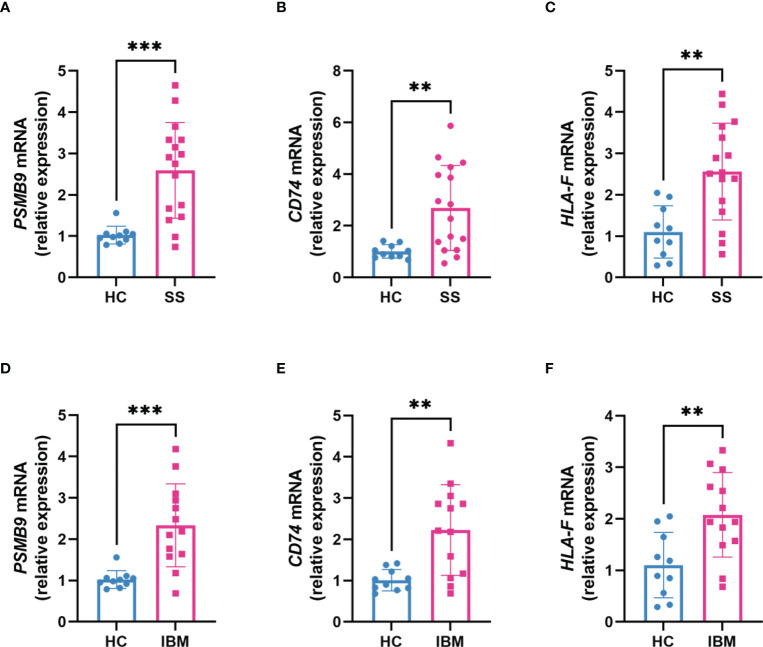
Transcription levels of hub genes in clinical SS and IBM patients. **(A–C)** The expression of *PSMB9*, *CD74* and *HLA-F* mRNA in SS patients (n=16) compared with healthy controls (n=10). **(D–F)** The expression of *PSMB9*, *CD74* and *HLA-F* mRNA in IBM patients (n=13) compared with healthy controls (n=10). The two groups were compared using a nonparametric Student’s t test with a *P* value of 0.05. ***p<0.001; **p<0.01; SS, Sjögren syndrome; IBM, inclusion body myositis.

To identify shared hub genes, we also analyzed the PPI networks using the Cytoscape plug-in cytoHubba. The top 20 genes were identified as potential hub genes using the MCC algorithm. Taking the intersection of the top 20 genes in the WGCNA and DEG sets, we identified three hub genes (*PSMB9*, *CD74*, and *HLA-F*) (**Figure 4**). Additionally, we conducted WGCNA analysis in GSE173808-PG and GSE128470 datasets and DEG analysis in GSE173808-LG and GSE151757 datasets. As shown in **Supplementary Figure 3**, 17 genes overlapped in the WGCNA and DEG algorithms, *PSMB9*, *CD74*, and *HLA-F* were also enriched, demonstrating strong consistency with our previous results (**Supplementary Figure 3J**) The expression levels of shared hub genes were further validated in the four datasets. Interestingly, in both SS and IBM, the expression of all hub genes was significantly higher than that in the control group. (**Figures 5A–D**). Furthermore, we evaluated the diagnostic power of the three hub genes across four datasets. Both GSE173808-LG and GSE173808-PG had area under the curve (AUC) values above 0.75, indicating their diagnostic value in SS. GSE151757 and GSE128470 both had AUC values > 0.95, demonstrating their high diagnostic value in IBM (**Figure 5E**). Further quantification of *PSMB9*, *CD74*, and *HLA-F* mRNA abundance revealed that these hub genes were actively transcribed in both SS (**Figures 6A–C**) and IBM patients (**Figures 6D–F**). In addition, the three genes (*PSMB9*, *CD74*, and *HLA-F*) were validated in other rheumatologic disorders including systemic lupus erythema (SLE) and rheumatoid arthritis (RA). Notably, only *HLA-F* expression in SLE and *CD74* expression in RA showed a statistically significant difference (**Supplementary Figures 4A–C**), and only *CD74* was found to be diagnostically significant in RA (AUC=0.93, **Supplementary Figures 4B–D**). Additionally, the expression levels and diagnostic values of the three genes were evaluated in other inflammatory myopathies, including PM, DM, and IMNM (**Supplementary Figure 5**). In PM, like IBM, all three genes were significantly upregulated and showed high diagnostic value, with an AUC >0.85 (**Supplementary Figures 5A, B**). However, only *PSMB9* and *HLA-F* expression in DM and *CD74* expression in IMNM showed statistically significant differences, which have certain diagnostic value for DM and IMNM (**Supplementary Figures 5C–F**).

### 3.4 Similar immune infiltration landscape between SS and IBM

**Figure 7:**
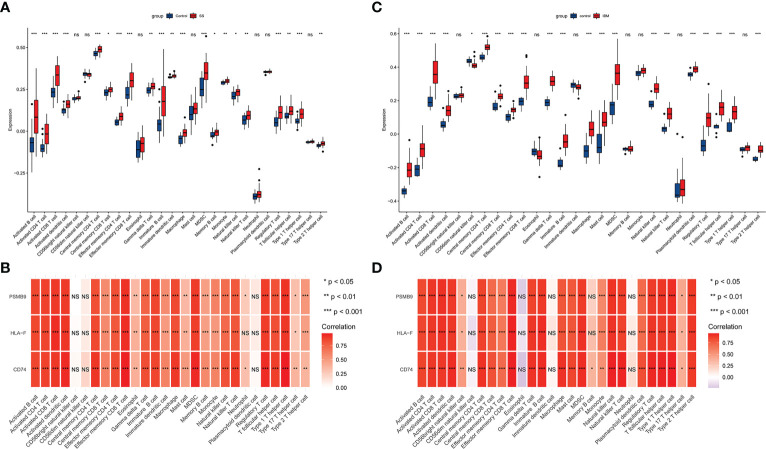
The immune infiltration landscape in SS and IBM and its association with hub genes. **(A)** Analysis of 28 types of immune cells in SS patients and controls with the ssGSEA algorithm. The blue box represents the control group, while the red box indicates the disease group. **(B)** Hub genes and immune cell infiltration heatmaps in SS. **(C)** Analysis of 28 types of immune cells in IBM patients and controls with the ssGSEA algorithm. **(D)** Hub genes and immune cell infiltration heatmaps in IBM. *p< 0.05; **p< 0.01; ***p< 0.001; ns, non-significant. SS, Sjögren syndrome; IBM, inclusion body myositis.

Several studies have suggested that IBM is a late complication of SS, so we hypothesized that IBM and SS may share common pathological mechanisms and similar immune microenvironments. With GSE173808-LG and GSE151757 datasets, we assess the extent of infiltration of 28 immune cell types using the ssGSEA algorithm. As shown in **Figures 7A–C**, SS and IBM demonstrated similar immune infiltration patterns, such as B cells, CD4 T cells, CD8 T cells, dendritic cells (DCs), myeloid-derived suppressor cells (MDSCs) and natural killer (NK) cells. All hub genes were positively associated with immune cell abundance (**Figures 7B–D**).

### 3.5 Prediction of TFs

**Figure 8:**
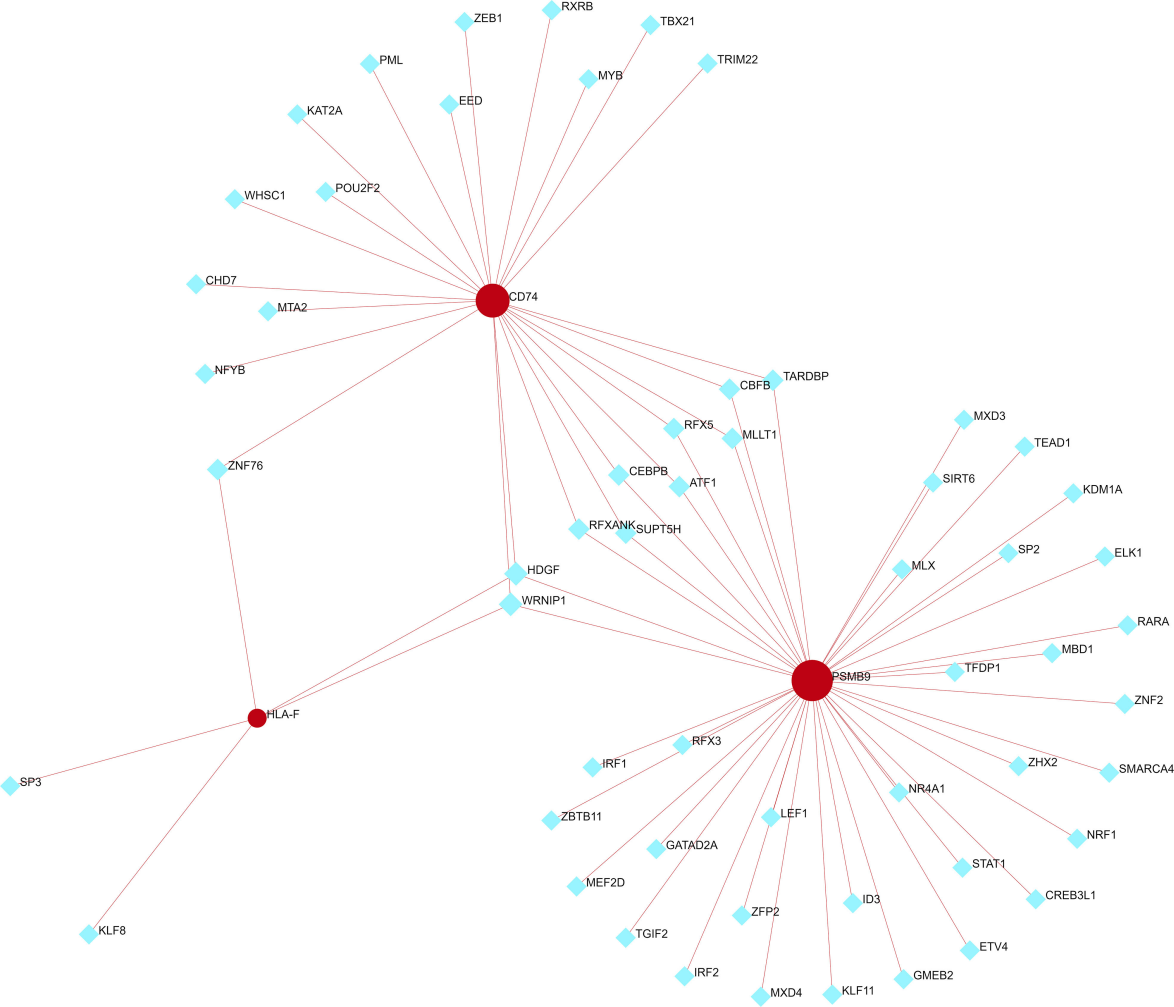
TF-shared hub gene regulatory network. The red circle represents a shared hub gene, while the blue diamond represents transcription factors. TFs, transcription factors.

We used NetworkAnalyst to predict the TFs that interact with the three shared hub genes and Cytoscape to visualize the TF-gene regulatory network. **Figure 8** shows that HDGF and WRNIP1 interact with all three hub genes, possibly regulating their expression. However, further research is needed to confirm these findings.

## 4 Discussion

IBM and SS are autoimmune diseases, and the two sometimes coexist. However, their common pathogenic mechanisms remain unclear despite intense research. As of now, clonal T expansion abnormalities (16, 24, 25), genetic predisposition linked to the MHC (23), and shared autoantibodies (18, 19) have been reported to be potential links between SS and IBM. However, few studies have examined the genetic link between SS and IBM. Our study is the first to integrate data from the GEO databank and use bioinformatic and experimental methods to investigate the common mechanisms between SS and IBM. Additionally, we found that both SS and IBM have identical immune infiltration microenvironments, strongly suggesting that similar immune responses are important for SS-IBM association.

### 4.1 Viral infection in SS and IBM pathogenesis

Global gene expression studies can provide us with a greater understanding of SS and IBM pathobiology. According to the functional enrichment of the WGCNA and DEG sets, we found that viral infection and antigen processing and presentation could play important roles in both SS and IBM.

Infections with viruses are closely related to SS and IBM. Epstein−Barr virus (EBV), a DNA virus, is capable of inducing autoimmune responses in SS (30, 31). Salivary glands from SS patients contained EBV antigens and viral DNA, as well as autoantigens homologous to EBV antigens, which suggests that EBV infection could contribute to autoimmune reactions through a molecular mimicry mechanism (31, 32). In addition, EBV can induce the production of type I interferon (IFN-I), which is a key factor in SS pathogenesis (33). Other viral infections, such as hepatitis C virus (HCV), cytomegalovirus (CMV) and human T lymphotropic virus type I (HTLV-1), are also associated with SS (34). HCV infection can exhibit SS symptoms and salivary gland lymphocytic infiltration (35). IgM antibodies against CMV were found to be higher in the serum of SS patients than controls (36), and HTLV-1 exhibited direct affinities for salivary glands according to Japanese studies (34, 37). Viral infection also plays a major role in IBM. For example, human immunodeficiency virus (HIV) and HTLV-1, two retroviruses, have been implicated in IBM (16). HIV-positive patients may develop an IBM-like phenotype with remarkable weakness of the finger flexors and knee extensors (38). Among 46 HIV-myopathy patients, three had typical IBM-like pathological features. Interestingly, 11 HTLV-1-positive patients showed a similar clinical and pathological phenotype to HIV-IBM (39). In addition, HCV, EBV, and influenza have been reported to be indirectly linked to IBM (16). It is speculated that the exhaustion of the immune system resulting from viral chronicity and antiviral medication may contribute to IBM muscle damage (40). In our study, functional enrichment analysis revealed EBV and HTLV-1 infections as common pathogens, so we hypothesized that both SS and IBM could be initiated by a common viral infection. The serum antibodies against EBV and HTLV-1 in patients with SS and IBM will need to be monitored in the future. Vaccines or drugs that block these viral infections may be effective targets for preventing SS and IBM.

### 4.2 Antigen processing and presentation in SS and IBM pathogenesis

Antigen processing and presentation are immunological processes by which whole antigens are fragmented and bound to the major histocompatibility complex (MHC) for presentation on the cell surface (41). There are two different antigen presentation pathways: MHC class I molecules present cytosolic antigens, whereas MHC class II molecules present extracellular proteins (41). In our analyses, antigen processing and presentation were the most significantly enriched KEGG terms, whether in the WGCNA algorithm or in DEG analysis. In addition, multiple alleles of MHC class I and II are enriched in most GO terms, indicating a key role for MHC molecules in SS and IBM.

The MHC is also known as human leukocyte antigen (HLA). Hundreds of genes reside within the HLA region and play fundamental roles in immunity. HLA alleles are the strongest heritable predictors of various autoimmune diseases, including SS and IBM (42, 43). The 6p21 locus carries three HLA alleles (*HLA-DRA*, *HLA-DQB1*, and *HLA-DQA1*) strongly associated with SS (44). In a Han Chinese population study, *HLA-DRB1/HLA-DQA1* and *HLA-DPB1/COLI1A2* at the 6p21.3 locus were identified as independent association signals with SS (45). Moreover, a meta-analysis by Cruz-Tapias et al. identified HLA-DQA1*05:01, HLA-DQB1*02:01 and HLA-DRB1*03:01 as risk factors for SS, while HLA-DQA1*02:01, HLA-DQA1*03:01 and HLA-DQB1*05:01 were protective factors for SS (46). Similar to SS, the strongest genetic risk associated with IBM lies within the HLA region, where HLA-DRB1 and HLA-DR3 are implicated (47–50). Moreover, Rojana-udomsart et al. reported six patients suffering from both SS and IBM, with all of them carrying the 8.1 ancestral haplotype and HLA-DR3 (23), suggesting a strong possibility that the coexistence of SS and IBM is linked to a common genetic predisposition associated with the MHC.

Our enrichment analysis results and the studies mentioned above also demonstrated a possible explanation for the coexistence of SS and IBM. A common viral infection triggers autoimmunity, and genetic susceptibility associated with the MHC leads to abnormalities in antigen processing and presentation, functioning in the pathogenesis of SS and IBM.

### 4.3 Shared key genes in SS and IBM

In this study, three shared hub genes (*PSMB9*, *CD74* and *HLA-F*) were obtained by integrating the WGCNA and DEG sets, and all three were significantly overexpressed in both the SS and IBM groups compared with the controls. The *PSMB9* gene is located within the MHC class II region and encodes a key component of the immunoproteasome complex, proteasome subunit beta type-9 (PSMB9) (51). The immunoproteasome, part of the ubiquitin−proteasome system, breaks down intracellular proteins into peptide fragments that bind to MHC molecules and triggers the presentation of antigens (51, 52). PSMB9 is also targeted by IFN-I, which induces immune activation and regulates proinflammatory cytokines and mediators (52). A variety of studies have demonstrated that PSMB9 dysregulation contributes to autoimmune diseases, such as SS (53), rheumatoid arthritis (54), systemic lupus erythematosus (SLE) (51) and dermatomyositis (51). In refractory SLE, the inhibition of PSMB9 with bortezomib antibody significantly improved symptoms by targeting plasma cells and type I interferons (55). Cluster of differentiation 74 (CD74), also called HLA class II histocompatibility antigen gamma chain, is a nonpolymorphic type II transmembrane glycoprotein that contributes to antigen presentation (56). CD74 can regulate immune cell function and development by interacting with macrophage migration inhibitory factor (MIF) (56). Inflammatory diseases such as ankylosing spondylitis, SLE and type I diabetes are associated with CD74 (56, 57). A recent bioinformatics study identified DEGs in IBM using the GSE39454 and GSE128470 datasets. They showed that CD74 is a hub gene, which is in consistent with our findings (58). Human leukocyte antigen F (HLA-F) is a nonclassical heavy chain that forms a complex with a β-2 microglobulin light chain (59). In contrast to many other HLA heavy chains, HLA-F is mainly found in the ER and Golgi apparatus rather than on the surface of most tissues (59). As an important immune regulator, HLA-F binds directly to immunostimulatory and immunoinhibitory receptors on immune cells such as NK cells and presents uncharacteristically long peptides to T cells (59, 60). Furthermore, HLA-F can trigger an NK cell response, preventing viral replication and killing infected cells (61). Higher expression of HLA-F and its genetic variants has been related to a predisposition to autoimmune diseases, such as SLE (62), rheumatoid arthritis (63) and ankylosing spondylitis (64). Taken together, these findings show that PSMB9, CD74, and HLA-F all play important roles in presenting antigens and triggering autoimmune diseases. It is likely that the dysregulation of these genes results in an imbalance in immunity and may contribute to the progression of SS and IBM. Furthermore, by building TF-hub gene networks, we discovered that HDGF and WRNIP1 interact with all three hub genes. However, few studies have elucidated the role of HDGF and WRNIP1 in SS and IBM, and their potential relationship needs further study. Futhermore, we analyzed the expression levels and diagnostic values of the three genes in other rheumatologic disorders and inflammatory myopathies. We observed prominent upregulation of all three genes in PM patients, with all of them exhibiting high diagnostic value. In contrast, only one or two genes were significantly diagnostic in other diseases. These results imply a plausible link between IBM and PM due to similar pathogenic molecular mechanisms. The overarching question remains whether PM and IBM are distinct disease entities or part of a shared spectrum of idiopathic inflammatory myopathies (IIMs) (16). When taken as a whole, these three hub genes serve as relatively specific biomarkers for SS and IBM, which may enable new breakthroughs in the areas of diagnosis and drug therapy.

### 4.4 Similar immune infiltration landscapes in SS and IBM

Since lymphocytic infiltration in corresponding tissues is a common pathological feature in SS and IBM, we used the ssGSEA algorithm to analyze the immune cell infiltration landscape in both diseases. A remarkable similarity was observed between SS and IBM in terms of immune infiltration. CD4 T cells, CD8 T cells, B cells, DCs, MDSCs, and NK cells were significantly upregulated in both groups. In line with our findings, previous studies have shown that SS is a lymphoproliferative disease with a predominance of T and B lymphocyte infiltration in salivary glands. A variety of NK cells and DCs are also present during the progression of SS (65). Similar scenarios have also been reported in IBM. In IBM muscle biopsy samples, CD8 T cells were detected in the endomysium, as well as macrophages (3, 66). Interestingly, abnormal clonal expansion of T cells in both SS and IBM was recently described (16, 24, 25). In light of the similarity of SS and IBM in terms of immune landscape and histopathology, we hypothesize that similar immune cell infiltration and disturbed immune homeostasis after viral infection in individuals with a genetic predisposition can contribute to the pathogenesis of SS and IBM.

In conclusion, the findings of our work suggest that viral infection and antigen processing and presentation dysregulation might be common susceptibility factors for the coexistence of SS and IBM. Three key genes (*PSMB9*, *CD74* and *HLA-F*) might be used as biomarkers or therapeutic targets in the future. Nevertheless, our study has a few limitations that need to be acknowledged. Firstly, we were unable to collect salivary gland tissue samples from SS patients, and as a result, blood specimens were used for validation instead. Secondly, SS and IBM are uncommon diseases, which makes it challenging to obtain tissue samples from patients with both conditions. Limited availability of such samples in the pathology library made it difficult to verify our bioinformatic findings. Future experimental studies with larger sample sizes should be conducted to elucidate the potential mechanisms of hub genes and related pathways.

## 5 Conclusion

In our study, we discovered that IBM shares similar immunologic and transcriptional pathways with SS, including viral infection and antigen processing/presentation. The three hub genes (*PSMB9*, *CD74* and *HLA-F*) might be relatively specific biomarkers for the two diseases. Additionally, both IBM and SS exhibit nearly identical immune infiltration microenvironments, potentially indicating that similar immune responses may be involved in their pathogenesis.

## Data availability statement

The datasets presented in this study can be found in online repositories. The names of the repository/repositories and accession number(s) can be found in the article/**Supplementary Material**.

## Ethics statement

The studies involving human participants were reviewed and approved by Ethics Committee of Sichuan Provincial People’s hospital. The patients/participants provided their written informed consent to participate in this study.

## Author contributions

LZ and QZ participated in the research design. LZ and KC participated in bioinformatic analysis. YW and LL performed the RT-qPCR experiment. FX, J-YB, C-YZ and ST collected clinical data and tissues from patients. LZ and QZ drafted the manuscript, YW and LL revised and polished the manuscript. All authors contributed to the article and approved the submitted version.
