# Supplementary material for: Potential common molecular mechanisms between Sjögren syndrome and inclusion body myositis: a bioinformatic analysis and in vivo validation
Source: Front Immunol. 2023 Apr 21;14:1161476. doi: 10.3389/fimmu.2023.1161476 (PMC10160489; doi:10.3389/fimmu.2023.1161476)
Supplement: Supplementary file 1 [file DataSheet_1.docx]

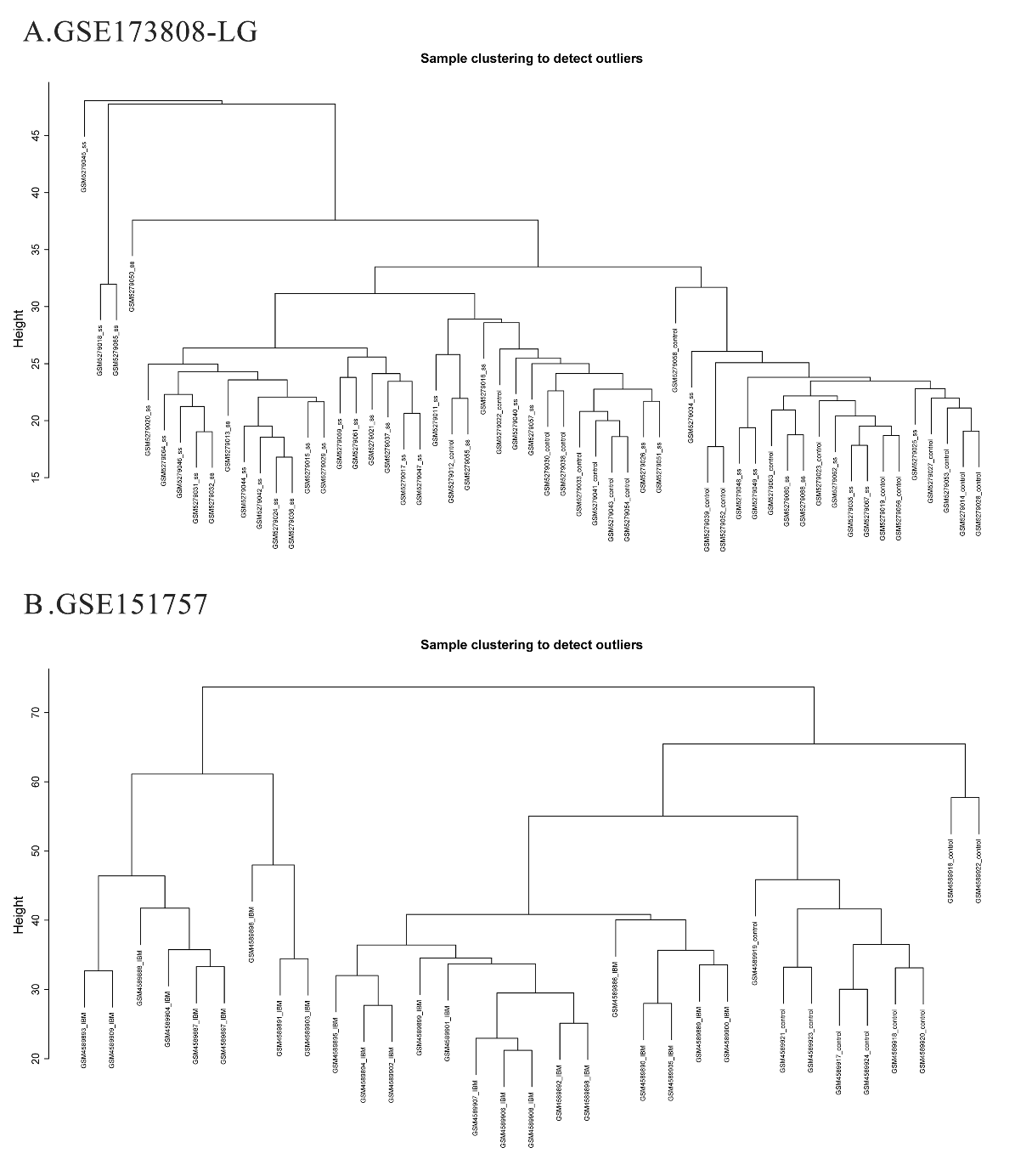


**Supplementary Figure 1.** Clustering dendrogram of SS and IBM datasets. In this graph, the abscissa corresponds to the sample name while the ordinate corresponds to the clustering degree. (A) GSE173808 dataset showing labial gland tissues from SS patients. (B) GSE151757 dataset showing skeletal muscle from IBM patients. SS, Sjögren syndrome; IBM, inclusion body myositis; LG, labial gland.
