# Supplementary material for: Potential common molecular mechanisms between Sjögren syndrome and inclusion body myositis: a bioinformatic analysis and in vivo validation
Source: Front Immunol. 2023 Apr 21;14:1161476. doi: 10.3389/fimmu.2023.1161476 (PMC10160489; doi:10.3389/fimmu.2023.1161476)
Supplement: Supplementary file 2 [file DataSheet_2.docx]

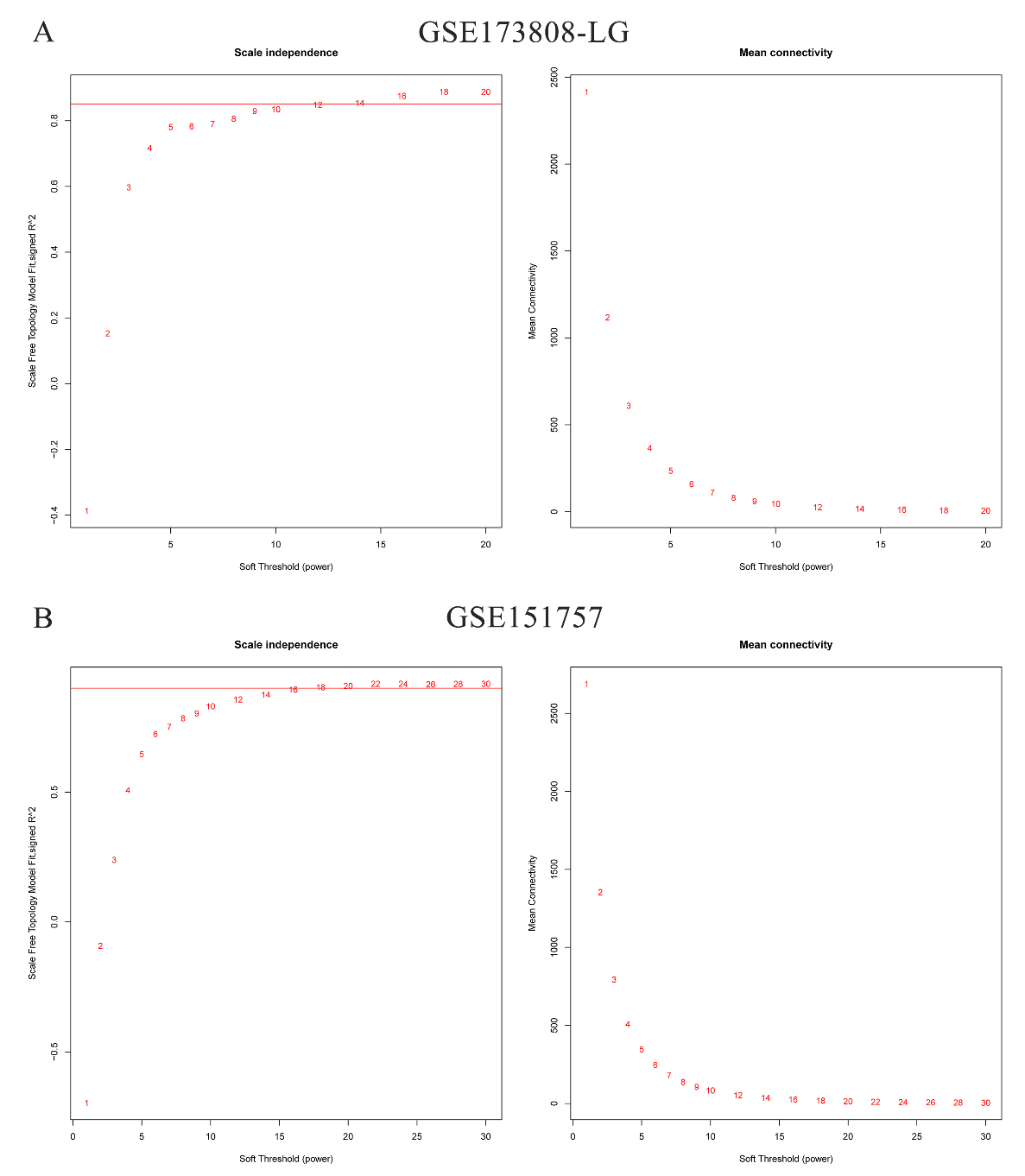


**Supplementary Figure 2.** Determination of soft threshold power in WGCNA analysis. The red line indicates that the square of correlation coefficient equals 0.9. (A) The approximate scale-free fit index can be obtained at the soft threshold power of 12 in GSE173808-LG dataset. (B) The approximate scale-free fit index can be obtained at the soft threshold power of 14 in GSE151757 dataset.
