# Supplementary material for: Potential common molecular mechanisms between Sjögren syndrome and inclusion body myositis: a bioinformatic analysis and in vivo validation
Source: Front Immunol. 2023 Apr 21;14:1161476. doi: 10.3389/fimmu.2023.1161476 (PMC10160489; doi:10.3389/fimmu.2023.1161476)
Supplement: Supplementary file 3 [file DataSheet_3.docx]

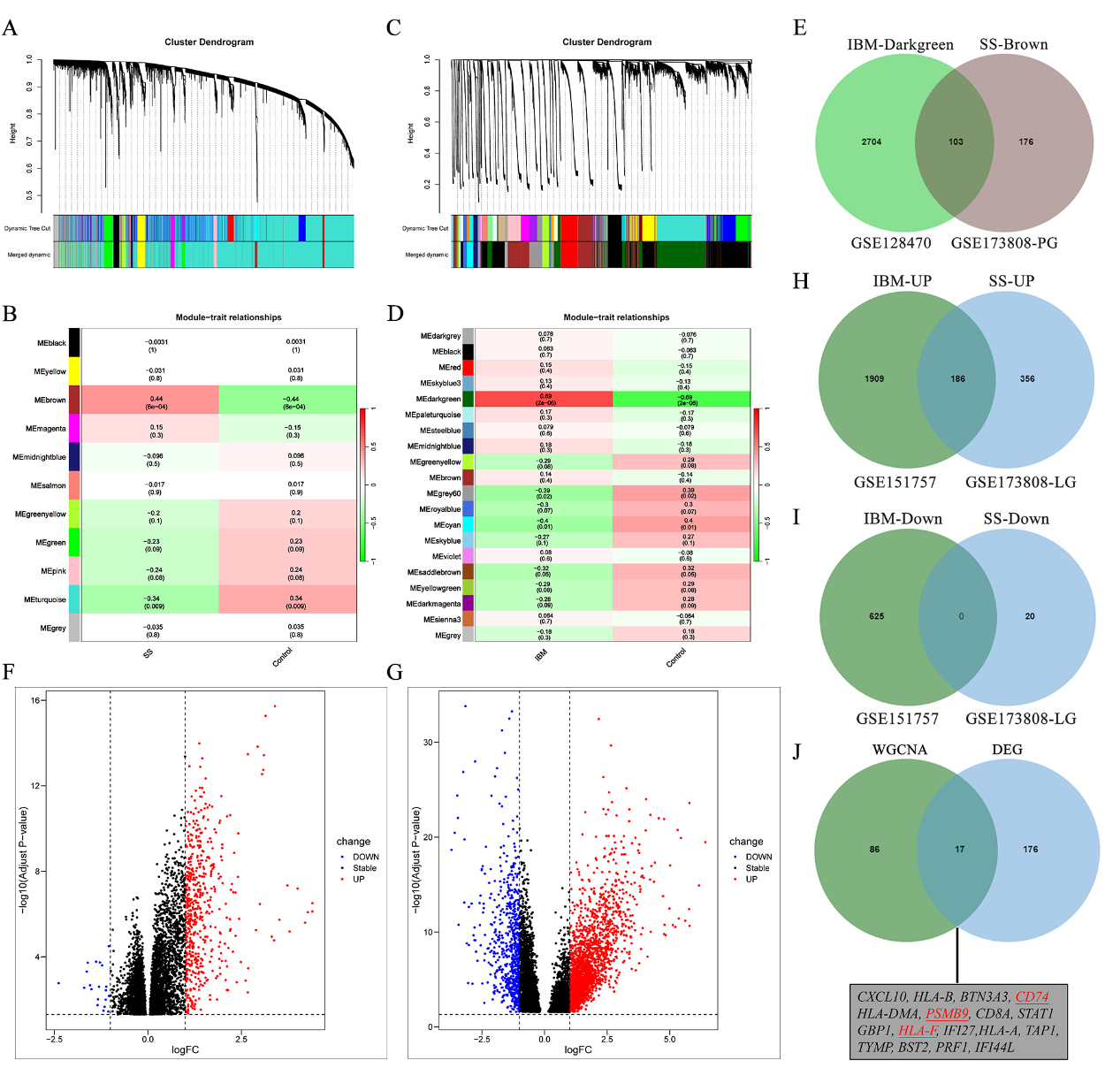


Supplementary Figure 3. Identification of shared genes in SS and IBM using WGCNA and DEG analysis. (A-D) WGCNA of the GSE173808-PG and GSE128470 datasets. (A) The coexpression gene cluster dendrogram in SS-PG (GSE173808-PG). (B) Correlation analysis between module genes and clinical phenotypes in SS-PG. (C) The coexpression gene cluster dendrogram in IBM (GSE128470). (D) Correlation analysis between module genes and clinical phenotypes in IBM. (E) Venn diagram for intersecting genes between the brown module of SS and the darkgreen module of IBM. (F-G) DEG analysis of the GSE173808-LG (F) and GSE151757 datasets (G). (H-I) Venn diagram for shared DEGs in the GSE173808-LG and GSE151757 datasets. (J) Venn diagram of common genes from the WGCNA and DEG analysis. WGCNA, weighted gene coexpression network analysis; DEGs, differentially expressed genes; SS, Sjögren syndrome; IBM, inclusion body myositis.
