# Supplementary material for: Potential common molecular mechanisms between Sjögren syndrome and inclusion body myositis: a bioinformatic analysis and in vivo validation
Source: Front Immunol. 2023 Apr 21;14:1161476. doi: 10.3389/fimmu.2023.1161476 (PMC10160489; doi:10.3389/fimmu.2023.1161476)
Supplement: Supplementary file 4 [file DataSheet_4.docx]

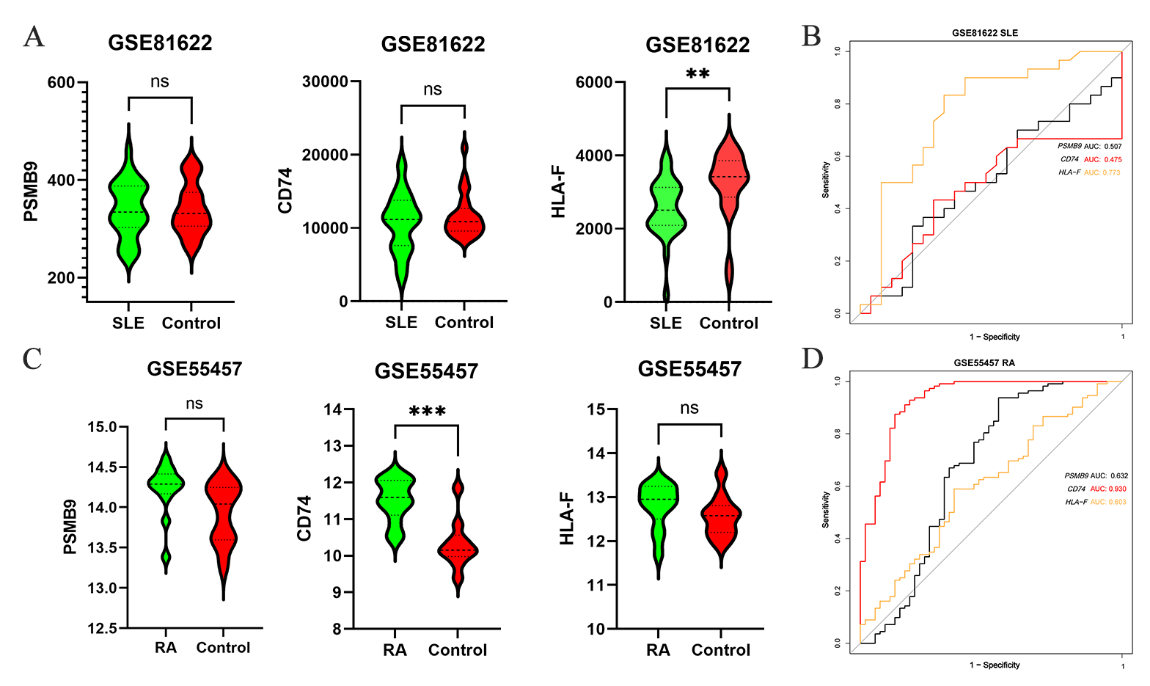


Supplementary Figure 4. Expression levels and diagnostic value of three hub genes in SLE and RA. (A) The expression levels of *PSMB9*, *CD74* and *HLA-F* in PBMC from patients with SLE (n=30) were compared with those of controls (n=25) using the GSE81622 dataset. (B) The potential diagnostic value of *PSMB9*, *CD74* and *HLA-F* in the SLE samples was investigated. (C) The expression levels of *PSMB9*, *CD74* and *HLA-F* in synovial membrane tissue from RA patients (n=13) were compared with those of controls (n=10) using the GSE55457 dataset. (D) The potential diagnostic value of *PSMB9*, *CD74* and *HLA-F* in the RA samples was evaluated. Comparisons between groups were performed using the nonparametric Student's t test, and a *P* value less than 0.05 was considered statistically significant. ***p*<0.01; ****p*<0.001; ns=non-significant. PBMC, peripheral blood mononuclear cell; SLE, systemic lupus erythema; RA, rheumatoid arthritis.
