# Supplementary material for: Potential common molecular mechanisms between Sjögren syndrome and inclusion body myositis: a bioinformatic analysis and in vivo validation
Source: Front Immunol. 2023 Apr 21;14:1161476. doi: 10.3389/fimmu.2023.1161476 (PMC10160489; doi:10.3389/fimmu.2023.1161476)
Supplement: Supplementary file 5 [file DataSheet_5.docx]

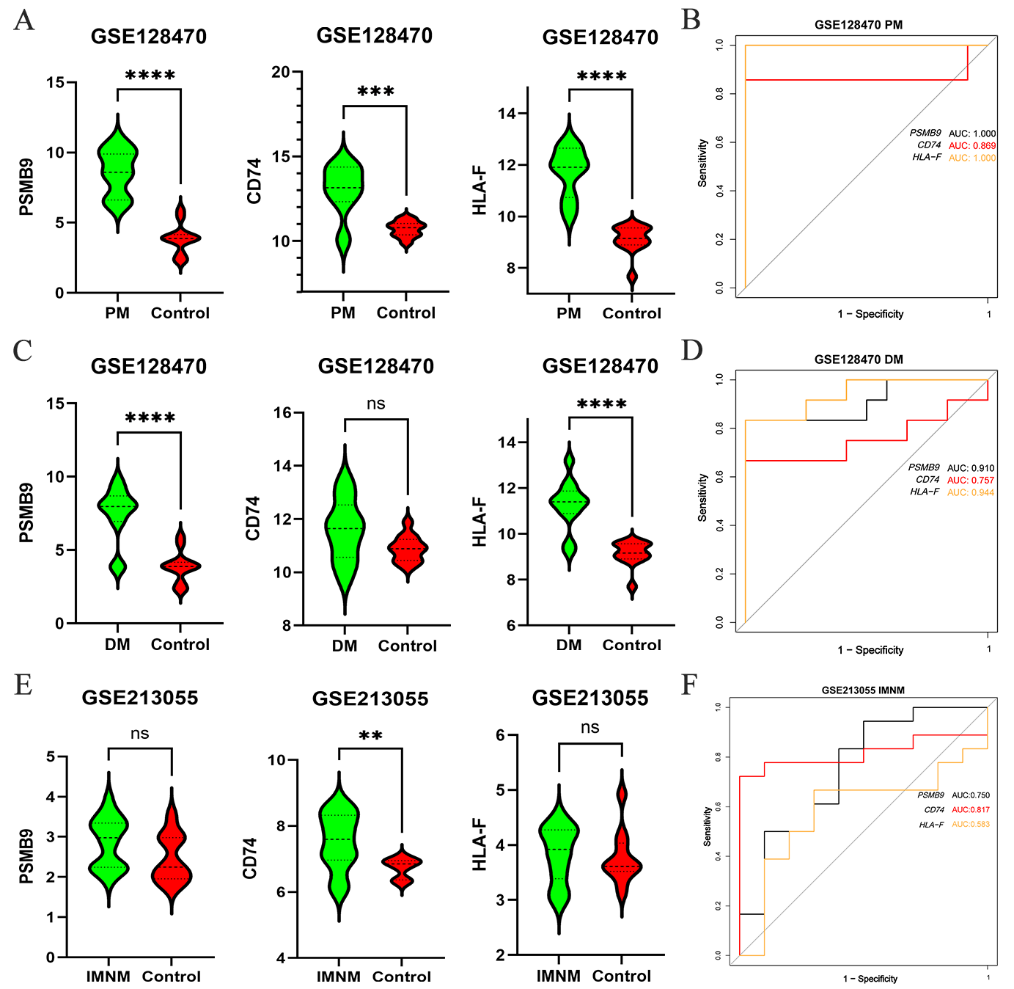


Supplementary Figure 5. Expression levels and diagnostic value of three hub genes in PM, DM, and INMN. (A) The expression levels of *PSMB9*, *CD74* and *HLA-F* in muscle from patients with PM (n=7) were compared with those of controls (n=12) using GSE128470 dataset. (B) The potential diagnostic value of *PSMB9*, *CD74* and *HLA-F* in the PM samples was investigated. (C) The expression levels of *PSMB9*, *CD74* and *HLA-F* in muscle from patients with DM (n=12) were compared with those of controls (n=12) using the GSE128470 dataset. (D) The potential diagnostic value of *PSMB9*, *CD74* and *HLA-F* in the DM samples was evaluated. (E) The expression levels of *PSMB9*, *CD74* and *HLA-F* in muscle from patients with IMNM (n=18) were compared with those of controls (n=10) using the GSE213055 dataset. (F) The potential diagnostic value of *PSMB9*, *CD74* and *HLA-F* in the IMNM samples was evaluated. Comparisons between groups were performed using the nonparametric Student's t test, and a *P* value less than 0.05 was considered statistically significant. ***p*<0.01; *****p*<0.0001; ns=non-significant. PM, polymyositis; DM, dermatomyositis; IMNM, immune-mediated necrotizing myopathy.
